# Allostatic load and progression of cardio-renal multimorbidity: A UK biobank study

**DOI:** 10.1371/journal.pone.0339576

**Published:** 2026-01-05

**Authors:** Qianshen Zhu, Lingling Xu, Zhixing Fan, Hongbo Li

**Affiliations:** 1 Department of Nephrology, Traditional Chinese and Western Medicine Hospital of Wuhan, Tongji Medical College, Huazhong University of Science and Technology, Wuhan, China; 2 Department of Hematology and Rheumatology, Wuhan Sixth Hospital Affiliated to Jianghan University, Wuhan, China; 3 Department of Cardiology, The First College of Clinical Medical Science, China Three Gorges University and Yichang Central People’s Hospital, Yichang, China; Dynamical Business & Science Society - DBSS International SAS, COLOMBIA

## Abstract

**Background:**

Cardio-renal multimorbidity (CRM), the coexistence of cardiovascular disease (CVD) and chronic kidney disease (CKD), imposes a significant healthcare burden. Allostatic load (AL), indicating cumulative physiological dysregulation from chronic stress, may be a modifiable risk factor for CRM.

**Methods:**

This study included 396,927 participants with a median follow-up of 13.67 years. AL was assessed via 10 biomarkers. Multistate models were used to analyze transitions from health to first cardio-renal disease (FCRD), to CRM, and to death.

**Results:**

Higher AL was significantly associated with increased risks of progression from health to FCRD, to CRM, and to death. The transition from FCRD to CRM was most affected by high AL. AL also had a stronger association with the transition from health to CKD than to CVD. Stratified analyses showed more pronounced associations in younger participants, those with higher socioeconomic status (SES), and unhealthy diets.

**Conclusion:**

AL is a significant upstream factor in CRM development and progression. Early identification of individuals with high AL could aid in risk assessment and prevention strategies for CRM.

## 1. Introduction

Cardio-renal multimorbidity (CRM) is defined as the coexistence of cardiovascular disease (CVD) and chronic kidney disease (CKD), two major conditions that frequently occur together and share complex pathophysiological mechanisms. Growing evidence has highlighted the nuanced and interdependent relationship between CVD and CKD, including shared hemodynamic phenotypes, similar underlying pathophysiological processes, and overlapping clinical outcomes, which often complicate the management and prognosis of both diseases [1,2]. As the global prevalence of CRM continues to rise, there is an increasing recognition of the substantial healthcare burden associated with these conditions, reflected in escalating healthcare expenditures [3,4]. The identification of novel and modifiable risk factors for CRM is therefore essential to improve prevention, early detection, and treatment strategies, ultimately alleviating the growing healthcare burden.

Allostatic load (AL) refers to the cumulative physiological wear and tear on the body resulting from chronic stress exposure and is considered a key indicator of stress-induced dysregulation [5]. AL is typically assessed through a set of biomarkers, including those that reflect cardiovascular, metabolic, and inflammatory systems [6]. Previous cross-sectional studies have demonstrated that higher levels of AL are associated with various adverse health outcomes, such as psychiatric disorders, cognitive decline, and chronic somatic conditions like CVD and CKD [7–9]. Moreover, AL has been proposed as a potential pathway linking susceptibility to both coronary heart disease (CHD) and CKD, with its effects mediated through differential mechanisms across these conditions [10–12]. However, while much attention has been given to the cross-sectional relationship between AL and individual health outcomes, the longitudinal association between AL and the progression of CRM remains underexplored.

To fill this gap, the present study aimed to investigate the prospective associations between AL and progression from health to FCRD, CRM and mortality using advanced polymorphic modelling, and to assess the differential impact of AL on specific disease transitions along the CRM continuum. Thus, deepening our understanding of the interactions between stress, physiological disorders and chronic disease progression, providing valuable insights for early intervention and improved risk stratification in clinical practice.

## 2. Methods

### 2.1. Study population

The UK Biobank received approval from the North West Multi-Centre Research Ethics Committee (Approval number: 21/NW/0157), the National Information Governance Board for Health and Social Care in England and Wales, and the Community Health Index Advisory Group in Scotland, with all participants providing written informed consent. This research utilized the UK Biobank Resource (Application ID: 170605). The UK Biobank is a national prospective cohort study that enrolled over 500,000 volunteer participants, aged 37–73, from England, Wales, and Scotland between March 2006 and October 2010, with multiple follow-ups conducted subsequently. A detailed description of the cohort has been documented elsewhere [13]. At the baseline, participants were requested to furnish socio-demographic details, behavioral characteristics, and health-related information. Blood samples were gathered for genotyping and biochemistry tests. Among the 502,175 participants with available data in the current study, those with CVD or CKD at baseline (diagnosed or self-reported, n = 64,697) were excluded. We then excluded participants with missing data on select covariates (N = 40,551). Finally, 396,927 participants were involved in the current study (**S1 Fig in S1 File**).

### 2.2. Follow-up for cardio-renal disease and death

Incident CVD and CKD, as well as relevant mortality, were ascertained from self-reported information, primary care data and hospital admission data. We utilized the corresponding International Classification of Diseases, 10th Revision (ICD10) or the Office of Population Censuses and Surveys Classification of Interventions and Procedures, version 4 (OPCS-4) codes to identify relevant diagnoses (S1 Table in S1 File). CVD was defined codes pertaining to coronary heart disease, atrial fibrillation, heart failure, peripheral artery disease, and stroke [14]. FCRD refereed to the first occurrence of any CVD or CKD during the follow-up period, representing the initial onset of cardio-renal impairment. CRM was defined as the coexistence of two CRDs after the initial FCRD, capturing the progression from single-organ to multi-organ involvement. These definitions reflect the clinically recognized trajectory from early cardio-renal dysfunction to multimorbidity, allowing systematic investigation of disease transitions.

### 2.3. Assessment of allostatic load

AL was evaluated based on 10 biomarkers that signify three physiological systems: metabolic, cardiovascular, and inflammatory, and all were assessed at the baseline [15]. In particular, the degree of metabolic dysregulation was measured through the quantification of serum glucose (mmol/L), total cholesterol (mmol/L), HDL cholesterol (mmol/L), HbA1c (mmol/mol), IGF-1 (nmol/L), waist–to-hip ratio, and body mass index (BMI, kg/m2) [16,17]. The level of inflammatory dysregulation was determined by CRP (mg/L), whereas cardiovascular dysregulation was gauged using systolic blood pressure (SBP; mmHg) and diastolic blood pressure (DBP; mmHg) [18]. Each biomarker was then dichotomized into high risk versus low risk in accordance with sex-specific quartiles [19,20]. The study established separate high-risk thresholds for male and female participants: serum glucose, total cholesterol, glycated haemoglobin, waist-to-hip ratio, BMI, C-reactive protein, insulin-like growth factor-1, systolic blood pressure, diastolic blood pressure exceeding the 75th percentile for their respective gender, or high-density lipoprotein cholesterol below the 25th percentile for their respective gender, were classified as high-risk for that indicator. Using these gender-specific cut-off values, participants received 1 point for each high-risk indicator met and 0 points for low-risk indicators. The detailed were listed in S2 Table in S1 File. The AL score was computed by summing the 10 dichotomous scores for each of the 10 markers, yielding a score ranging from 0 to 10, where higher scores denoted a more pronounced physical dysregulation. Subsequently, a 3-group variable was considered in line with previous studies, with low (0–2), mid (3–4), and high (5–10) AL [21].

### 2.4. Covariates

The covariates comprised age (continuous, years), ethnicity (white or other), educational level (College or University, A levels/AS level or equivalent, O levels/GCSE or equivalent, CSEs or equivalent, NVQ/HND/HNC or equivalent, other professional qualifications), socioeconomic standing (measured by Townsend deprivation index, TDI), BMI (categorized as < 18.5 kg/m^2^, 18.5–24.9 kg/m^2^, 25.0–29.9 kg/m^2^, or ≥30 kg/m^2^), smoking status (never, previous, current), alcohol intake status (never, previous, current), dietary patterns (categorized as a healthy diet with ≥4 scores or an unhealthy diet with < 4 scores in accordance with recent guidelines for optimal cardiovascular health) [22].

### 2.5. Statistical analysis

Categorical variables were presented by counts (percentages), while continuous variables were depicted using either the mean (standard deviation, SD) or the median (interquartile range, IQR). The survival time for each participant was computed as the period from the baseline to the date of the incident, death, or censoring (31 October 2020), whichever came first. Two multivariable-adjusted models were established: Model 1 was adjusted for age, and ethnicity; Model 2 was adjusted for age, ethnicity, BMI, education level, TDI, dietary patterns, smoking status, and alcohol intake status.

We initially employed the Cox regression model to investigate the correlations of AL with FCRD, CRM, and death respectively. Schoenfeld residuals detected no significant violation of the proportional hazard assumptions. we then utilized multistate models to assess the association between AL and longitudinal progression from healthy to FCRD, then to CRM, and ultimately to death. There were five transitions among the three states: (A) health to FCRD, (B) health to death from a disease other than CVD, and CKD, (C) FCRD to CRM, (D) FCRD to death from any cause and (E) CRM to death from any cause. For those participants who entered different states on the same date, we determined the entry date of the theoretical prior state as the entry date of the latter state minus 0.5 days. Based on transition pattern A, we further contemplated the specific types of FCRD in disease progression. Seven transitions between five states were considered: (A) health to CVD, (B) health to CKD, (C) health to death from a disease other than CVD, and CKD, (D) CVD to CRM, (E) CKD to CRM, (F) CVD to death from any cause, (G) CVD to death from any cause and (H) CRM to death from any cause. Additionally, we further explored the potential mediating effects of WBC count and NEUT count in the association of AL with FCRD, CRM, and all-cause death using counterfactual mediation analysis.

We further conducted a series of sensitivity analyses to ensure the robustness of our results. First, we performed stratified analyses based on age, TDI, dietary patterns, smoking status and alcohol intake. Second, we repeated the analyses with complete data, employed random forest imputation for missing data, generated distinct imputed datasets (m = 5) to account for uncertainty, and set a random seed (seed = 500) for reproducibility. Third, cases of cardiovascular disease, chronic kidney disease and death occurring within two years were excluded to minimise potential reverse causation. Fourth, we replicated the analysis after excluding participants with cancer at baseline to alleviate the impact of a typically shortened lifespan linked to cancer. Fifth, we duplicated the analysis using age as the time scale to account for the influence of age on survival time. Sixth, for those participants who entered diverse states on the same day, we calculated the entry date of the prior state by employing different time intervals (30, 180, and 360 days) to assess the influence of these intervals on the outcomes. Seventh, we re-ran the analyses after omitting the cardiovascular domain from the AL when evaluating CVD risk to avoid potential statistical bias. Eighth, we reconstructed the AL score using alternative cut-points based on established clinical thresholds to test the stability of our findings across different definitions [23].

All analyses were conducted using R version 4.3.0 (R Project). The multistate model was performed using the “mstate” package. The mediation analysis was conducted using the “CMAverse” package. The Missing data were imputation using the “mice” package. *P* < 0.05 was considered statistically significant.

## 3. Results

### 3.1. Characteristics of participants

**Fig 1:**
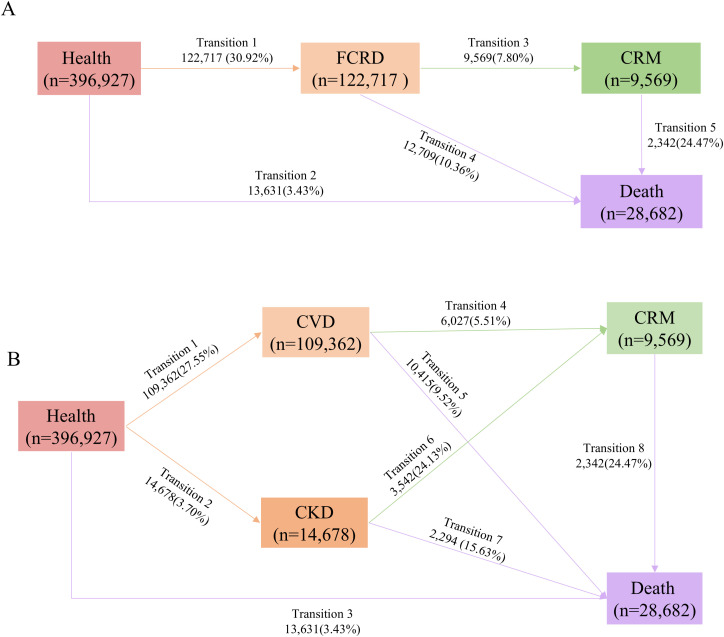
Numbers and percentages of participants across disease transitions: (A) from health to FCRD, CRM, and death; (B) from health to specific FCRD (CVD or CKD), followed by CRM, and ultimately to death. Abbreviations: FCRD, first cardio-renal disease; CRM, cardio-renal multimorbidity; CRMM, cardio-renal-metabolic multimorbidity; CVD, cardiovascular disease; CKD, chronic kidney disease.

Among 396,927 participants, the mean age was 55.95 (SD = 8.07) years, 169,786 (42.96%) participants were male, and 377,437 (95.09%) were white ethnicity. Participants with higher AL tended to be men and older, have higher body mass index, less vulnerable socioeconomic status (SES), more like to smoke, and favor access alcohol intake (Table 1). In addition, during a median follow-up of 13.67 (IQR = 12.93 to 14.39) years, 122,717 (30.92%) participants experienced FCRD. Of all CRDs patients, 9,569 (7.80%) developed CRM, and, afterwards, 2,342 (24.47%) died from CRM. Additionally, 13,631 (3.43%) died without experiencing CRDs (Fig 1A). In terms of specific disease transitions, 109,362 (27.55%) incident cases of CVD, and 14,678 (3.70%) incident cases of CKD (Fig 1B). The distribution of data variables was shown in S2 Fig in S1 File.

**Table 1:** Baseline characteristics of study participants by incident disease status during follow-up.

Characteristics	AL				*P*
	Total (n = 396,927)	Low (n = 218,089)	Mid (n = 130,183)	High (n = 48,655)	
Age, years	55.95 (8.07)	54.87 (8.20)	56.89 (7.84)	58.27 (7.26)	<0.001
Male (%)	169,786 (42.96)	92,677 (42.76)	56,420 (43.44)	20,689 (42.57)	<0.001
White (%)	377,237 (95.09)	207,824 (95.29)	123,446 (94.82)	45,967 (94.48)	<0.001
BMI, kg/m^2^	27.22 (4.71)	25.65 (3.69)	28.44 (4.95)	30.92 (5.06)	<0.001
TDI	−1.47 (2.99)	−1.52 (2.97)	−1.43 (3.01)	−1.34 (3.03)	<0.001
College degree (%)	138,417 (34.88)	83,513 (38.29)	41,593 (31.95)	13,311 (27.36)	<0.001
Smoking statues (%)					<0.001
Never	223,609 (56.35)	126,586 (58.04)	71,486 (54.91)	25,537 (52.49)	
Previous	133,373 (33.62)	69,368 (31.81)	45,476 (34.93)	18,529 (38.08)	
Current	39,945 (10.04)	22,135 (10.15)	13,221 (10.16)	4,589 (9.43)	
Alcohol intake status (%)					<0.001
Never	15,256 (3.82)	8,087 (3.71)	5,093 (3.91)	2,076 (4.27)	
Previous	12,202 (3.06)	6,657 (3.05)	4,116 (3.16)	1,429 (2.94)	
Current	369,469 (93.12)	203,345 (93.24)	120,974 (92.93)	45,150 (92.80)	
Healthy diet (%)	192,624 (48.52)	104,630 (47.98)	63,468 (48.75)	24,526 (50.41)	<0.001

BMI: calculated as weight in kilograms divided by the square of height in meters (kg/m2); TDI: Townsend Deprivation Index, a standardized, unitless composite measure of area-level socioeconomic deprivation, with higher values indicating greater deprivation; CVD: cardiovascular disease, FCRD: first cardio-renal disease, CRM: cardio-renal multimorbidity.

### 3.2. AL and longitudinal progression of CRM

**Fig 2:**
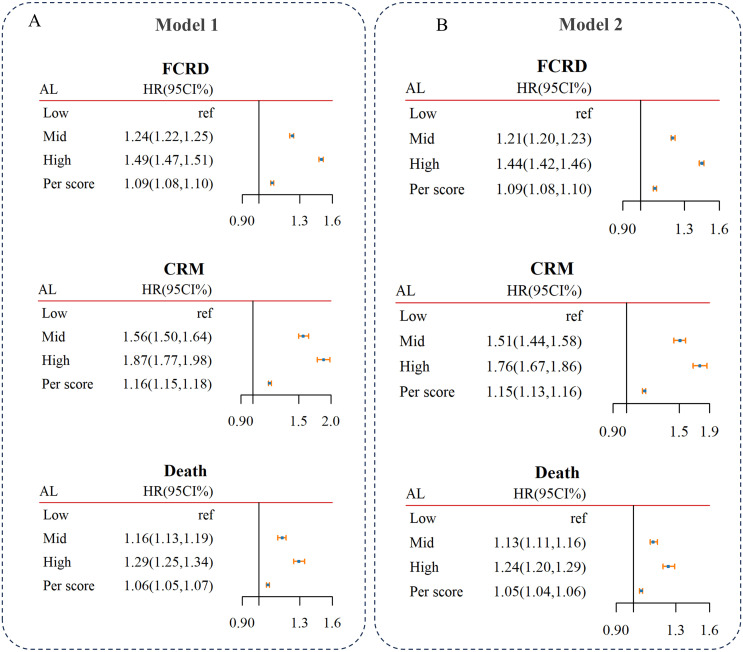
Associations of AL with the risks of FCRD, CRM, and death using Cox model. Models 2 were adjusted for age, ethnicity, BMI, education level, TDI, physical activity, dietary patterns, smoking status, alcohol intake status. Models1 were adjusted for age, ethnicity. HR: hazard ratio, CI: confidence interval, TDI: Townsend deprivation index, AL: Allostatic load, FCRD: first cardio-renal disease, CRMM: cardio-renal multimorbidity.

Cox analysis revealed that a high of AL was associated with higher odds of FCRD, CRM and death (Fig 2). The multistate analysis further indicated that a high AL was associated with the elevated risk of all transitions from healthy to FCRD, to CRM and to death, particularly the transition from FCRD to CRM, followed by transition from health to FCRD. The influence of AL was broadly similar for the transitions from FCMD to Death and from CRMM to Death. Furthermore, participants with higher AL demonstrated an augmented risk of progression to CRDs and death compared to those with medium or low AL (Table 2).

**Table 2:** Multistate model to assess association of AL with progression from health to FCMD, then to CRMM, and to death.

Model 2	AL			
	Low	Mid	High	Per score
**Health to FCRD**	**ref**	1.21(1.2,1.23)*	1.44(1.42,1.46)*	1.09(1.08,1.09)*
**Health to Death**	**ref**	1.04(1.01,1.08)*	1.00(0.95,1.06)	1.01(1.00,1.02)
**FCRD to CRM**	**ref**	1.37(1.31,1.43)*	1.54(1.46,1.63)*	1.11(1.10,1.13)*
**FCRD to Death**	**ref**	1.09(1.05,1.14)*	1.27(1.21,1.33)*	1.06(1.05,1.07)*
**CRM to Death**	**ref**	1.13(1.03,1.24)*	1.28(1.15,1.43)*	1.06(1.03,1.08)*
**Model 1**	**AL**			
	**Low**	**Mid**	**High**	**Per score**
**Health to FCRD**	**ref**	1.24(1.22,1.25)*	1.49(1.47,1.51)*	1.1(1.09,1.10)*
**Health to Death**	**ref**	1.06(1.03,1.10)*	1.03(0.98,1.08)	1.01(1.00,1.02)
**FCRD to CRM**	**ref**	1.41(1.35,1.47)*	1.61(1.52,1.70)*	1.12(1.11,1.14)*
**FCRD to Death**	**ref**	1.11(1.07,1.16)*	1.31(1.24,1.37)*	1.06(1.05,1.08)*
**CRM to Death**	**ref**	1.13(1.03,1.25)*	1.29(1.16,1.45)*	1.06(1.04,1.09)*

Note: Models 2 were adjusted for age, ethnicity, BMI, education level, TDI, physical activity, dietary patterns, smoking status, alcohol intake status. Models 1 were adjusted for age, ethnicity. TDI: Townsend deprivation index, AL: Allostatic load, FCRD: first cardio-renal disease, CRM: cardio-renal multimorbidity, CVD: cardiovascular disease, CKD: chronic kidney disease.

### 3.3. AL and disease-specific transitions of CRM

**Fig 3:**
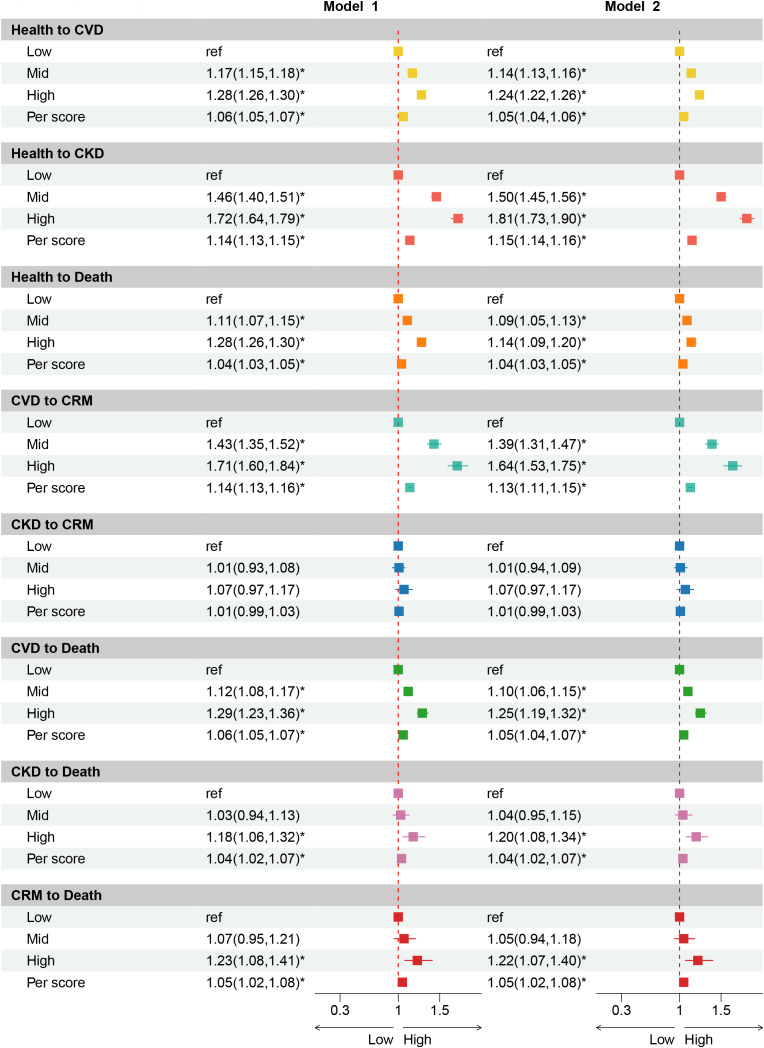
Association of AL with progression from health to specific FCMD (CVD, and CKD), then to CRMM, and to death using multistate model. Model 2 were adjusted for age, ethnicity, education level, TDI, dietary patterns, smoking status, alcohol intake status. Model 1 were adjusted for age, ethnicity. HR: hazard ratio, TDI: Townsend deprivation index, FCRD: first cardio-renal disease, CRM: cardio-renal multimorbidity.

We further probed into the specific types of FCRD in the dynamic progression of the disease (Transition Pattern B). It was discovered that, exception of the transition from CKD to CRM, AL exerts a remarkable influence on other transition states. Specifically, the association between AL and the transition from health to CVD as well as from CKD to CRM was the most intimate. In comparison to the transition from healthy to CVD, a higher AL was more prone to boost the risk of the transition from health to CKD. Nevertheless, the impact of AL on the transition from CVD to death was more substantial than that from CKD to death. Additionally, in contrast to individuals with medium or low AL, participants with high AL possessed a higher risk of contracting CVD and CKD (Fig 3).

### 3.4. Stratified analysis and sensitivity analysis

The AL manifested more pronounced correlations with the advancement among participants aged 45–50 years, those with a high SES, and individuals adhering to an unhealthy diet, as opposed to older participants, those with a low SES, and individuals with a healthy diet **(S3-S4 Tables in** S1 File). After calculating covariates **(S5 Table in S1 File**, excluding outcome events that occurred during the two-year follow-up period **(S6 Table in** S1 File), and excluding patients with cancer at baseline **(S7 Table in** S1 File), there was little change in the results. Even when age was employed as the time scale and diverse time intervals were implemented for participants transitioning into different states on the same date, the robust associations between AL and the progression risk of CRDs remained conspicuous **(S8-S9 Tables in** S1 File). Moreover, omitting the cardiovascular domain when evaluating CVD risk **(S10 Table in** S1 File) and reconstructing the AL score with alternative clinical cut-points yielded concordant results, supporting the robustness of our findings **(S11–S12 Tables in** S1 File).

## 4. Discussion

In this study, we found that elevated AL was significantly associated with increased risks of developing FCRD, progressing to CRM, and experiencing subsequent mortality. These associations remained robust across multistate models and sensitivity analyses, reinforcing the hypothesis that chronic stress burden contributes to both the onset and acceleration of cardio-renal disease trajectories.

Among the transitions examined, the most pronounced effect of high AL was observed in the shift from FCRD to CRM. This suggests that AL may not only function as a trigger for the initial onset of disease but also exacerbate the vulnerability to multimorbidity once a single organ system is compromised [24–26]. Elevated AL appears to accelerate the transition to more severe stages of disease, particularly in the context of cardiovascular and renal health [25,27–30]. This acceleration was likely due to the cumulative wear and tear on the body’s physiological systems, which could impair the body’s ability to maintain homeostasis and resilience against further health challenges [31,32]. Moreover, AL was positively associated with transitions from CRM to death, further emphasizing its long-term impact across the entire continuum of disease progression [33,34]. These findings highlight the critical role of AL as a key factor in disease exacerbation and progression, reinforcing the notion that physiological stress accumulation may accelerate the onset and worsening of multimorbidity over time.

Our study builds upon and extends prior work by demonstrating the dynamic, rather than static, role of AL in the development of multimorbidity, shifting the focus from singular disease outcomes to the intricate relationships among multiple conditions. By utilizing a multistate model framework, we were able to capture the complexity of disease transitions over time and uncover the differential effects of AL across various stages of progression. Specifically, elevated AL was associated with an increased incidence of both cardiovascular and renal diseases, with a stronger association observed for the transition from health to CKD than to CVD [35,36]. This suggests a potential renal-specific sensitivity to systemic stress load [11]. Notably, our AL measure, consistent with many large-scale epidemiological studies [37,38], did not include neuroendocrine markers and therefore primarily reflects cardiometabolic and inflammatory dysregulation rather than the full spectrum of allostatic load. Consequently, the stronger association with CKD likely reflects downstream physiological effects captured by metabolic, cardiovascular, and inflammatory markers, rather than primary stress-mediated pathways. Stratified analyses revealed that the associations between AL and CRM outcomes were more pronounced in younger individuals, those with higher SES, and those adhering to unhealthy dietary patterns. These findings suggest that AL may serve as an often-hidden or underestimated risk factor, particularly within subpopulations traditionally regarded as lower risk by conventional models.

This study has several strengths. It leveraged a large, well-characterized cohort with long-term follow-up, enabling detailed assessment of rare but clinically important transitions such as CRM to death. The AL score used incorporated validated biomarkers spanning metabolic, cardiovascular, and inflammatory domains, reflecting the multisystem nature of stress burden. The multistate model provided a nuanced view of sequential disease development, allowing inference beyond binary outcomes. Nonetheless, several limitations should be noted. AL was assessed only at baseline, limiting insight into temporal fluctuations or cumulative exposure over time. Residual confounding from unmeasured stressors may exist despite extensive covariate adjustment. Importantly, our AL index did not include neuroendocrine markers (e.g., cortisol, catecholamines) due to unavailability, thus primarily reflecting cardiometabolic and inflammatory dysregulation and potentially underestimating the role of primary stress pathways in kidney function. Future studies incorporating neuroendocrine biomarkers are warranted. Moreover, the UK Biobank cohort is predominantly of European ancestry and healthier than the general population, which may limit the generalizability of our findings. Lastly, disease definitions based on ICD codes may under-detect early or subclinical cases.

## 5. Conclusion

In conclusion, our findings reinforce the role of AL as a modifiable upstream determinant of cardio-renal multimorbidity. Elevated AL was independently associated with increased risks of disease onset, progression to multimorbidity, and premature mortality. These results underscore the importance of early identification of individuals with elevated physiological stress, especially in the absence of clinical symptoms, and highlight the potential value of incorporating AL into future risk stratification and prevention strategies for multimorbidity.

## Supporting information

S1 File Supporting information could be found in Supplementary. (DOCX)
