# Supplementary material for: Allostatic load and progression of cardio-renal multimorbidity: A UK biobank study
Source: PLoS One. 2026 Jan 5;21(1):e0339576. doi: 10.1371/journal.pone.0339576 (PMC12768364; doi:10.1371/journal.pone.0339576)
Supplement: S1 File — (DOCX) [file pone.0339576.s001.docx]

**Supplementary**


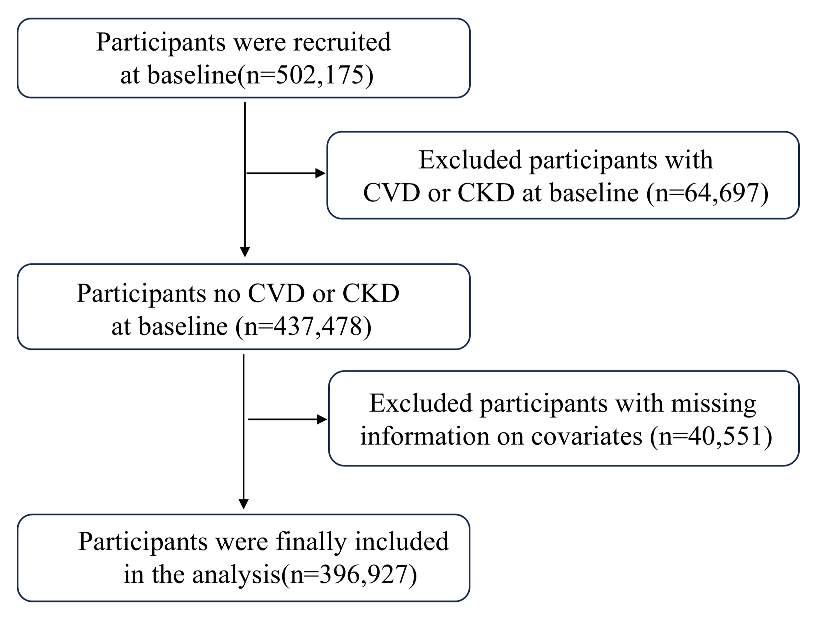


S1 Fig Flowchart of UK Biobank participants included for final analysis


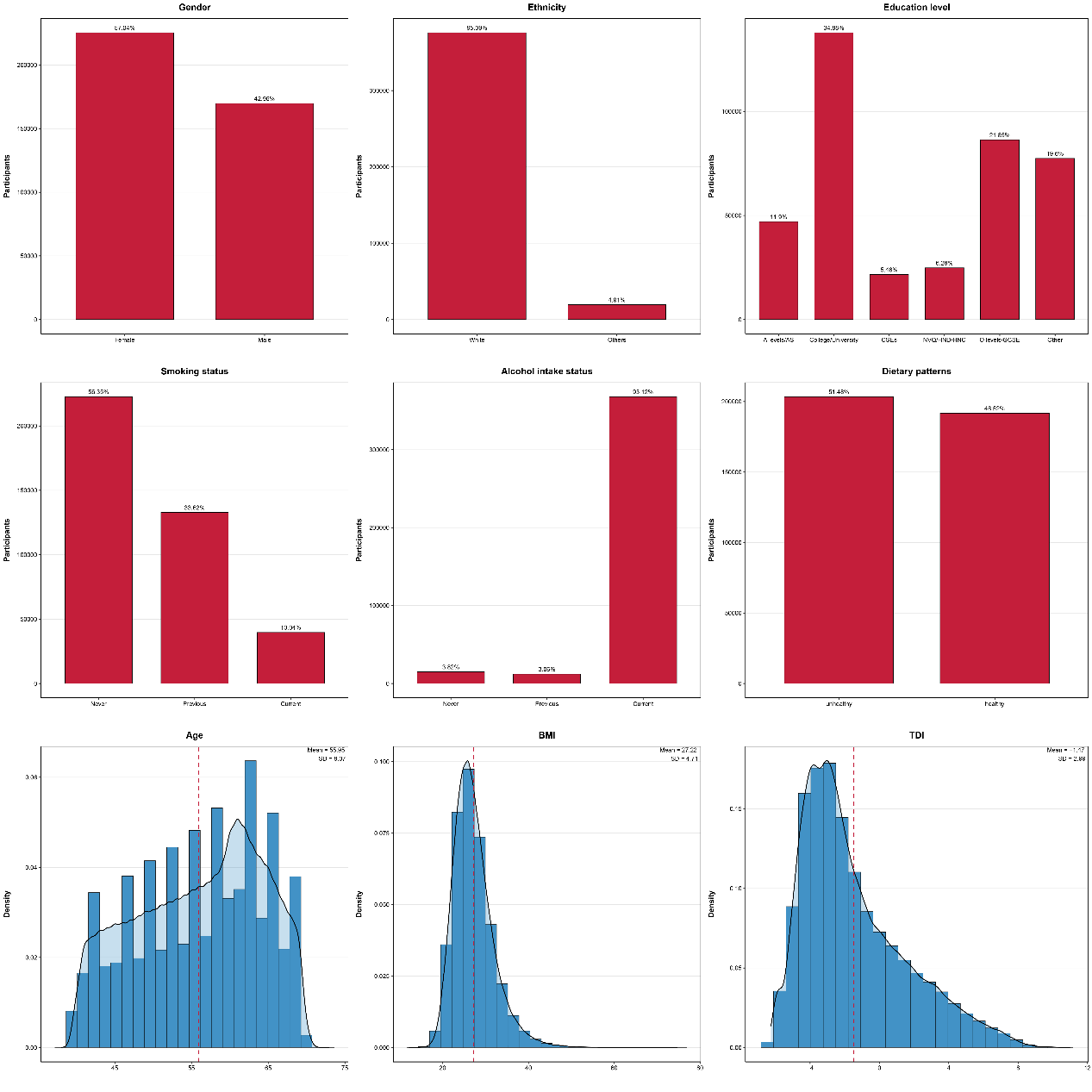


S2 Fig The distribution of data variables

**S1 Table. Ascertain the outcomes via Inter****national Classification of Disease and procedure codes**

| **Outcome** | **ICD or procedural codes** |
| --- | --- |
| CVD | ICD-10:  I11.0, I20, I20.0, I20.1, I20.8, I20.9, I21, I21.0, I21.1, I21.2, I21.3, I21.4, I21.9, I22, I22.0, I22.1, I22.8, I22.9, I23, I23.0, I23.1, I23.2, I23.3, I23.4, I23.5, I23.6, I23.8, I24, I24.0, I24.1, I24.8, I24.9, I25, I25.0, I25.1, I25.2, I25.5, I25.6, I25.8, I25.9, I42.0, I42.1, I42.5, I42.8, I42.9, I48, I49, I49.0, I49.1, I49.2, I49.3, I49.4, I49.5, I49.8, I49.9, I50, I50.0, I50.1, I50.9, I70.0, I70.00, I70.01, 170.2, 170.20, I70.21, I70.8, I70.80, I70.9, I70.90, I73.8, I73.9  OPCS-4:  K40, K40.1, K40.2, K40.3, K40.4, K40.8, K40.9, K41, K41.1, K41.2, K41.3, K41.4, K41.8, K41.9, K42, K42.1, K42.2, K42.3, K42.4, K42.8, K42.9, K43, K43.1, K43.2, K43.3, K43.4, K43.8, K43.9, K44, K44.1, K44.2, K44.8, K44.9, K45, K45.1, K45.2, K45.3, K45.4, K45.5, K45.6, K45.8, K45.9, K46, K46.1, K46.2, K46.3, K46.4, K46.5, K46.8, K46.9, K47, K47.1, K47.2, K47.3, K47.4, K47.5, K47.8, K47.9, K48.1, K48.2, K48.3, K48.4, K48.8, K48.9, K49, K49.1, K49.2, K49.3, K49.4, K49.8, K49.9, K50, K50.1, K50.2, K50.3, K50.4, K50.8, K50.9, K57.1, K62.1, K62.2, K62.3, K62.4, K63, K75, X09.3, X09.4, X09.5, L21.6, L51.3, L51.6, L51.8, L52.1, L52.2, L54.1, L54.4, L54.8, L59.1, L59.2, L59.3, L59.4, L59.5, L59.6, L59.7, L59.8, L60.1, L60.2, L63.1, L63.5, L63.9, L66.7, X50.1, X50.2 |
| CKD | ICD-10: N18, N18.0, N18.1, N18.2, N18.3, N18.4, N18.5, N18.8, N18.9  OPCS-4: M01, M01.1, M01.2, M01.3, M01.4, M01.5, M01.8, M01.9 |

Note: CVD: cardiovascular disease, CKD: chronic kidney disease, ICD: International Classification of Disease, OPCS-4: Office of Population Censuses and Surveys Classification of Interventions and Procedures, version 4.

**S2 Table. The details of allostatic load assessment.**

| **Domain** | **Variables** | **Field ID** | **Cut-points** | |
| --- | --- | --- | --- | --- |
|  |  |  | Male | Female |
| Metabolic | Glucose (mmol/L) | 30740 | 5.36 | 5.28 |
|  | Cholesterol | 30690 | 6.22 | 6.59 |
|  | HDL cholesterol | 30760 | 1.06 | 1.33 |
|  | Glycated haemoglobin (HbA1c) | 30750 | 38.10 | 37.70 |
|  | IGF-1 | 30770 | 25.20 | 24.40 |
|  | Waist circumference/Hip circumference | 48,49 | 0.98 | 0.96 |
|  | BMI (kg/m) | 21002,12144 | 30.00 | 29.70 |
| Inflammatory | C-reactive protein (mg/L) | 30170 | 2.53 | 2.96 |
| Cardiovascular | Systolic blood pressure (SBP, mmHg) | 4080 | 152.00 | 147.00 |
|  | Diastolic blood pressure (DBP, mmHg) | 4079 | 90.50 | 87.00 |

**S3 Table The results of stratified analyses by age.**

| **Subgroups** | **AL** | | | |
| --- | --- | --- | --- | --- |
|  | **Low** | **Mid** | **High** | **Per score** |
| **Age (years)** |  |  |  |  |
| **<45** |  |  |  |  |
| **Health to FCRD** | **ref** | 1.36(1.3,1.43) | 2.08(1.93,2.23) | 1.16(1.15,1.18) |
| **Health to Death** | **ref** | 1.15(0.97,1.37) | 1.55(1.18,2.05) | 1.08(1.03,1.14) |
| **FCRD to CRM** | **ref** | 1.32(0.94,1.85) | 1.87(1.19,2.95) | 1.15(1.05,1.26) |
| **FCRD to Death** | **ref** | 0.98(0.73,1.32) | 1.97(1.38,2.8) | 1.14(1.06,1.23) |
| **CRM to Death** | **ref** | 1.52(0.51,4.47) | 0.54(0.11,2.73) | 1(0.76,1.31) |
| **45-50** |  |  |  |  |
| **Health to FCRD** | **ref** | 1.42(1.36,1.47) | 1.91(1.81,2.02) | 1.16(1.15,1.17) |
| **Health to Death** | **ref** | 1.18(1.03,1.36) | 1.62(1.33,1.98) | 1.11(1.07,1.16) |
| **FCRD to CRM** | **ref** | 1.49(1.18,1.88) | 1.65(1.2,2.26) | 1.15(1.08,1.23) |
| **FCRD to Death** | **ref** | 1.36(1.13,1.65) | 1.55(1.2,2.01) | 1.1(1.05,1.16) |
| **CRM to Death** | **ref** | 0.81(0.38,1.7) | 3.62(1.63,8.06) | 1.23(1.01,1.49) |
| **51-55** |  |  |  |  |
| **Health to FCRD** | **ref** | 1.26(1.22,1.31) | 1.62(1.55,1.69) | 1.11(1.1,1.12) |
| **Health to Death** | **ref** | 1.09(0.98,1.21) | 1.07(0.91,1.25) | 1.03(1,1.06) |
| **FCRD to CRM** | **ref** | 1.53(1.3,1.81) | 1.65(1.34,2.04) | 1.13(1.08,1.18) |
| **FCRD to Death** | **ref** | 1.3(1.14,1.5) | 1.47(1.23,1.75) | 1.09(1.05,1.13) |
| **CRM to Death** | **ref** | 0.92(0.6,1.42) | 1.56(0.94,2.58) | 1.08(0.96,1.22) |
| **56-60** |  |  |  |  |
| **Health to FCRD** | **ref** | 1.18(1.15,1.21) | 1.46(1.41,1.52) | 1.09(1.08,1.1) |
| **Health to Death** | **ref** | 1.12(1.03,1.21) | 1.08(0.96,1.21) | 1.03(1,1.05) |
| **FCRD to CRM** | **ref** | 1.46(1.3,1.63) | 1.61(1.41,1.85) | 1.13(1.09,1.16) |
| **FCRD to Death** | **ref** | 1.21(1.1,1.33) | 1.44(1.29,1.62) | 1.08(1.06,1.11) |
| **CRM to Death** | **ref** | 1.09(0.83,1.41) | 1.34(0.99,1.82) | 1.08(1.01,1.16) |
| **61-65** |  |  |  |  |
| **Health to FCRD** | **ref** | 1.16(1.14,1.19) | 1.31(1.28,1.35) | 1.07(1.06,1.07) |
| **Health to Death** | **ref** | 1.01(0.95,1.08) | 0.93(0.85,1.02) | 0.99(0.97,1.01) |
| **FCRD to CRM** | **ref** | 1.36(1.26,1.47) | 1.63(1.49,1.79) | 1.12(1.1,1.14) |
| **FCRD to Death** | **ref** | 1.08(1.01,1.15) | 1.2(1.11,1.3) | 1.05(1.03,1.07) |
| **CRM to Death** | **ref** | 1.2(1.02,1.41) | 1.2(1,1.45) | 1.04(1,1.09) |
| **>65** |  |  |  |  |
| **Health to FCRD** | **ref** | 1.15(1.12,1.18) | 1.26(1.22,1.31) | 1.06(1.05,1.06) |
| **Health to Death** | **ref** | 0.97(0.9,1.04) | 0.89(0.81,0.99) | 0.98(0.96,1) |
| **FCRD to CRM** | **ref** | 1.31(1.21,1.41) | 1.4(1.27,1.53) | 1.09(1.07,1.12) |
| **FCRD to Death** | **ref** | 0.99(0.92,1.06) | 1.17(1.07,1.27) | 1.04(1.02,1.06) |
| **CRM to Death** | **ref** | 1.11(0.96,1.28) | 1.3(1.1,1.54) | 1.06(1.02,1.1) |

Note: Models2 were adjusted for age, ethnicity, BMI, education level, TDI, physical activity, dietary patterns, smoking status, alcohol intake status. Models1 were adjusted for age, ethnicity. TDI: Townsend deprivation index, AL: Allostatic load, FCRD: first cardio-renal disease, CRM: cardio-renal multimorbidity, CVD: cardiovascular disease, CKD: chronic kidney disease.

**S4 Table The results of** **stratified analyses by TDI, dietary patterns, smoking status and alcohol intake.**

| **Subgroups** | **AL** | | | |
| --- | --- | --- | --- | --- |
|  | **Low** | **Mid** | **High** | **Per score** |
| **TDI** |  |  |  |  |
| **Low** |  |  |  |  |
| **Health to FCRD** | **ref** | **1.22(1.20,1.24) *** | **1.46(1.43,1.49) *** | **1.09(1.08,1.09) *** |
| **Health to Death** | **ref** | **1.08(1.02,1.14) *** | **1.01(0.93,1.09) *** | **1.01(0.99,1.03) *** |
| **FCRD to CRM** | **ref** | **1.35(1.27,1.45) *** | **1.59(1.47,1.72) *** | **1.12(1.10,1.14) *** |
| **FCRD to Death** | **ref** | **1.15(1.09,1.22) *** | **1.33(1.24,1.44) *** | **1.07(1.05,1.09) *** |
| **CRM to Death** | **ref** | 1.00(0.87,1.15) | **1.24(1.05,1.46) *** | **1.07(1.03,1.11) *** |
| **High** |  |  |  |  |
| **Health to FCRD** | **ref** | **1.28(1.26,1.30) *** | **1.56(1.52,1.59) *** | **1.11(1.10,1.11) *** |
| **Health to Death** | **ref** | **1.09(1.04,1.15) *** | **1.11(1.03,1.19) *** | **1.02(1.01,1.04) *** |
| **FCRD to CRM** | **ref** | **1.42(1.33,1.51) *** | **1.57(1.46,1.70) *** | **1.11(1.10,1.13) *** |
| **FCRD to Death** | **ref** | **1.08(1.02,1.14) *** | **1.27(1.20,1.36) *** | **1.06(1.05,1.07) *** |
| **CRM to Death** | **ref** | **1.21(1.07,1.37) *** | **1.28(1.10,1.49) *** | **1.04(1.01,1.08) *** |
| **Diet** |  |  |  |  |
| **Healthy** |  |  |  |  |
| **Health to FCRD** | **ref** | **1.27(1.24,1.29) *** | **1.50(1.46,1.53) *** | **1.10(1.09,1.10) *** |
| **Health to Death** | **ref** | **1.11(1.05,1.17) *** | **1.10(1.02,1.19) *** | **1.03(1.01,1.04) *** |
| **FCRD to CRM** | **ref** | **1.39(1.30,1.48) *** | **1.58(1.46,1.71) *** | **1.12(1.10,1.14) *** |
| **FCRD to Death** | **ref** | **1.14(1.08,1.20) *** | **1.35(1.26,1.45) *** | **1.07(1.06,1.09) *** |
| **CRM to Death** | **ref** | 1.13(0.99,1.29) | **1.21(1.04,1.42) *** | **1.05(1.02,1.09) *** |
| **unhealthy** |  |  |  |  |
| **Health to FCRD** | **ref** | **1.24(1.22,1.26) *** | **1.52(1.48,1.55) *** | **1.10(1.09,1.10) *** |
| **Health to Death** | **ref** | **1.06(1.01,1.12) *** | 1.02(0.94,1.09) | 1.01(1.00,1.03) |
| **FCRD to CRM** | **ref** | **1.38(1.29,1.47) *** | **1.56(1.45,1.69) *** | **1.11(1.10,1.13) *** |
| **FCRD to Death** | **ref** | **1.08(1.03,1.15) *** | **1.25(1.17,1.34) *** | **1.06(1.04,1.07) *** |
| **CRM to Death** | **ref** | 1.09(0.96,1.25) | **1.31(1.13,1.53) *** | **1.06(1.02,1.09) *** |
| **Smoking** |  |  |  |  |
| **Never** |  |  |  |  |
| **Health to FCRD** | **ref** | **1.24(1.22,1.26) *** | **1.52(1.48,1.55) *** | **1.10(1.09,1.10) *** |
| **Health to Death** | **ref** | **1.09(1.03,1.15) *** | **1.14(1.06,1.24) *** | **1.03(1.01,1.05) *** |
| **FCRD to CRM** | **ref** | **1.40(1.31,1.50) *** | **1.65(1.52,1.80) *** | **1.13(1.11,1.15) *** |
| **FCRD to Death** | **ref** | **1.13(1.06,1.20) *** | **1.33(1.23,1.44) *** | **1.07(1.05,1.09) *** |
| **CRM to Death** | **ref** | 1.00(0.86,1.16) | 1.19(0.99,1.42) | **1.06(1.02,1.10) *** |
| **Previous** |  |  |  |  |
| **Health to FCRD** | **ref** | **1.26(1.24,1.29) *** | **1.49(1.45,1.52) *** | **1.10(1.09,1.10)** ***** |
| **Health to Death** | **ref** | **1.10(1.03,1.16) *** | 0.98(0.90,1.07) | 1.01(0.99,1.03) |
| **FCRD to CRM** | **ref** | **1.34(1.25,1.44) *** | **1.48(1.36,1.61) *** | **1.10(1.08,1.12)** ***** |
| **FCRD to Death** | **ref** | **1.08(1.02,1.15) *** | **1.38(1.28,1.48) *** | **1.08(1.06,1.09)** ***** |
| **CRM to Death** | **ref** | **1.23(1.07,1.41) *** | **1.38(1.18,1.63) *** | **1.06(1.03,1.10)** ***** |
| **Current** |  |  |  |  |
| **Health to FCRD** | **ref** | **1.27(1.22,1.32)** ***** | **1.55(1.48,1.63)** ***** | **1.10(1.09,1.11)** ***** |
| **Health to Death** | **ref** | 1.07(0.98,1.16) | 1.04(0.92,1.19) | 1.01(0.99,1.04) |
| **FCRD to CRM** | **ref** | **1.48(1.30,1.69)** ***** | **1.67(1.42,1.96)** ***** | **1.14(1.10,1.17)** ***** |
| **FCRD to Death** | **ref** | **1.15(1.06,1.26)** ***** | **1.04(0.93,1.18)** ***** | **1.03(1.01,1.05)** ***** |
| **CRM to Death** | **ref** | 1.09(0.87,1.36) | 1.16(0.88,1.53) | 1.03(0.97,1.09) |
| **Drinking** |  |  |  |  |
| **Never** |  |  |  |  |
| **Health to FCRD** | **ref** | **1.31(1.23,1.39)** ***** | **1.67(1.55,1.80)** ***** | **1.12(1.10,1.14)** ***** |
| **Health to Death** | **ref** | 1.07(0.89,1.29) | 1.12(0.88,1.44) | 1.03(0.98,1.08) |
| **FCRD to CRM** | **ref** | **1.50(1.24,1.81)** ***** | **1.64(1.31,2.07)** ***** | **1.11(1.06,1.17)** ***** |
| **FCRD to Death** | **ref** | 1.08(0.89,1.31) | **1.50(1.20,1.87)** ***** | **1.09(1.04,1.15)** ***** |
| **CRM to Death** | **ref** | 1.07(0.74,1.54) | 0.93(0.59,1.46) | 0.97(0.88,1.07) |
| **Previous** |  |  |  |  |
| **Health to FCRD** | **ref** | **1.36(1.28,1.45)** ***** | **1.65(1.52,1.79)** ***** | **1.12(1.11,1.14)** ***** |
| **Health to Death** | **ref** | 1.16(0.98,1.36) | 0.86(0.66,1.13) | 0.98(0.93,1.03) |
| **FCRD to CRM** | **ref** | **1.51(1.25,1.82)** ***** | **1.44(1.13,1.85)** ***** | **1.11(1.06,1.17)** ***** |
| **FCRD to Death** | **ref** | 1.04(0.88,1.23) | 1.09(0.88,1.36) | 1.02(0.98,1.07) |
| **CRM to Death** | **ref** | 1.15(0.80,1.66) | 0.86(0.53,1.38) | 1.01(0.92,1.11) |
| **Current** |  |  |  |  |
| **Health to FCRD** | **ref** | **1.24(1.23,1.26)** ***** | **1.49(1.47,1.52)** ***** | **1.10(1.09,1.10)** ***** |
| **Health to Death** | **ref** | **1.08(1.04,1.12)** ***** | **1.06(1.01,1.12)** ***** | **1.02(1.01,1.03)** ***** |
| **FCRD to CRM** | **ref** | **1.37(1.31,1.44)** ***** | **1.58(1.49,1.68)** ***** | **1.12(1.11,1.13)** ***** |
| **FCRD to Death** | **ref** | **1.12(1.07,1.16)** ***** | **1.30 (1.24,1.37)** ***** | **1.06(1.05,1.08)** ***** |
| **CRM to Death** | **ref** | **1.11(1.01,1.23)** ***** | **1.33(1.18,1.49)** ***** | **1.07(1.04,1.09)** ***** |

Note: Models2 were adjusted for age, ethnicity, BMI, education level, TDI, physical activity, dietary patterns, smoking status, alcohol intake status. Models1 were adjusted for age, ethnicity. TDI: Townsend deprivation index, AL: Allostatic load, FCRD: first cardio-renal disease, CRM: cardio-renal multimorbidity, CVD: cardiovascular disease, CKD: chronic kidney disease.

**S5 Table Association between AL and disease progression after** **interpolation of missing values for RF**

|  | **AL** | | | |
| --- | --- | --- | --- | --- |
|  | **Low** | **Mid** | **High** | **Per score** |
| **Health to FCRD** | **ref** | **1.21(1.20,1.23)** | **1.43(1.41,1.45)** | **1.09(1.08,1.09)** |
| **Health to Death** | **ref** | **1.03(1.00,1.07)** | 0.97(0.92,1.02) | 1.00(0.99,1.01) |
| **FCRD to CRM** | **ref** | **1.39(1.33,1.45)** | **1.55(1.47,1.63)** | **1.11(1.10,1.13)** |
| **FCRD to Death** | **ref** | **1.09(1.06,1.13)** | **1.26(1.20,1.32)** | **1.05(1.04,1.06)** |
| **CRM to Death** | **ref** | **1.14(1.05,1.24)** | **1.26(1.14,1.40)** | **1.06(1.04,1.08)** |

Note: Models2 were adjusted for age, ethnicity, BMI, education level, TDI, physical activity, dietary patterns, smoking status, alcohol intake status. Models1 were adjusted for age, ethnicity. TDI: Townsend deprivation index, AL: Allostatic load, FCRD: first cardio-renal disease, CRM: cardio-renal multimorbidity, CVD: cardiovascular disease, CKD: chronic kidney disease.

**S6 Table Association between AL and disease progression when age as time scales for in the** **multi-state model.**

|  | **AL** | | | |
| --- | --- | --- | --- | --- |
|  | **Low** | **Mid** | **High** | **Per score** |
| **Health to FCRD** | **ref** | 1.21(1.20,1.23) | 1.44(1.41,1.46) | 1.09(1.08,1.09) |
| **Health to Death** | **ref** | 1.05(1.01,1.09) | 1.01(0.96,1.06) | 1.01(1.00,1.02) |
| **FCRD to CRM** | **ref** | 1.37(1.31,1.44) | 1.55(1.47,1.64) | 1.11(1.10,1.13) |
| **FCRD to Death** | **ref** | 1.09(1.05,1.14) | 1.27(1.21,1.33) | 1.06(1.05,1.07) |
| **CRM to Death** | **ref** | 1.13(1.03,1.24) | 1.28(1.15,1.43) | 1.06(1.03,1.08) |

Note: Models2 were adjusted for age, ethnicity, BMI, education level, TDI, physical activity, dietary patterns, smoking status, alcohol intake status. Models1 were adjusted for age, ethnicity. TDI: Townsend deprivation index, AL: Allostatic load, FCRD: first cardio-renal disease, CRM: cardio-renal multimorbidity, CVD: cardiovascular disease, CKD: chronic kidney disease.

**S7 Table Association between AL and disease progression after exclusion of CKD and CVD occurring during the first 2 years of follow-up.**

|  | **AL** | | | |
| --- | --- | --- | --- | --- |
|  | **Low** | **Mid** | **High** | **Per score** |
| **Health to FCRD** | **ref** | 1.22(1.2,1.240) | 1.47(1.44,1.50) | 1.09(1.09,1.09) |
| **Health to Death** | **ref** | 1.04(1.00,1.08) | 0.99(0.94,1.05) | 1.00(0.99,1.02) |
| **FCRD to CRM** | **ref** | 1.37(1.31,1.44) | 1.55(1.46,1.64) | 1.11(1.10,1.13) |
| **FCRD to Death** | **ref** | 1.10(1.06,1.15) | 1.29(1.22,1.36) | 1.06(1.05,1.07) |
| **CRM to Death** | **ref** | 1.16(1.04,1.29) | 1.33(1.18,1.51) | 1.07(1.04,1.10) |

Note: Models2 were adjusted for age, ethnicity, BMI, education level, TDI, physical activity, dietary patterns, smoking status, alcohol intake status. Models1 were adjusted for age, ethnicity. TDI: Townsend deprivation index, AL: Allostatic load, FCRD: first cardio-renal disease, CRM: cardio-renal multimorbidity, CVD: cardiovascular disease, CKD: chronic kidney disease.

**S8 Table Association between AL and disease progression when exclusion participants who had cancer at baseline.**

|  | **AL** | | | |
| --- | --- | --- | --- | --- |
|  | **Low** | **Mid** | **High** | **Per score** |
| **Health to FCRD** | **ref** | 1.22(1.20,1.23) | 1.45(1.42,1.47) | 1.09(1.08,1.09) |
| **Health to Death** | **ref** | 1.04(1.00,1.09) | 1.00(0.95,1.06) | 1.01(1.00,1.02) |
| **FCRD to CRM** | **ref** | 1.39(1.32,1.45) | 1.53(1.44,1.63) | 1.11(1.10,1.13) |
| **FCRD to Death** | **ref** | 1.10(1.05,1.15) | 1.30(1.23,1.37) | 1.06(1.05,1.07) |
| **CRM to Death** | **ref** | 1.12(1.01,1.24) | 1.24(1.10,1.41) | 1.05(1.03,1.08) |

Note: Models2 were adjusted for age, ethnicity, BMI, education level, TDI, physical activity, dietary patterns, smoking status, alcohol intake status. Models1 were adjusted for age, ethnicity. TDI: Townsend deprivation index, AL: Allostatic load, FCRD: first cardio-renal disease, CRM: cardio-renal multimorbidity, CVD: cardiovascular disease, CKD: chronic kidney disease.

**S9 Table Association between AL and disease progression when using different** **intervals for the participants entering different states on the same date.**

| **Intervals** | **AL** | | | |
| --- | --- | --- | --- | --- |
|  | **Low** | **Mid** | **High** | **Per score** |
| **30 days** |  |  |  |  |
| **Health to FCRD** | **ref** | **1.25(1.24,1.27) *** | **1.51(1.48,1.53)** ***** | **1.10(1.09,1.10)** ***** |
| **Health to Death** | **ref** | **1.09(1.05,1.13)** ***** | **1.06(1.01,1.12)** ***** | **1.02(1.01,1.03)** ***** |
| **FCRD to CRM** | **ref** | **1.39(1.32,1.45)** ***** | **1.58(1.49,1.67)** ***** | **1.12(1.10,1.13)** ***** |
| **FCRD to Death** | **ref** | **1.11(1.07,1.16)** ***** | **1.30(1.24,1.36)** ***** | **1.06(1.05,1.07)** ***** |
| **CRM to Death** | **ref** | **1.11(1.01,1.22)** ***** | **1.27(1.14,1.41)** ***** | **1.06(1.03,1.08)** ***** |
| **180 days** |  |  |  |  |
| **Health to FCRD** | **ref** | **1.25(1.24,1.27)** ***** | **1.51(1.48,1.53)** ***** | **1.10(1.09,1.10)** ***** |
| **Health to Death** | **ref** | **1.09(1.05,1.13)** ***** | **1.06(1.01,1.12)** ***** | **1.02(1.01,1.03)** ***** |
| **FCRD to CRM** | **ref** | **1.38(1.32,1.45)** ***** | **1.58(1.49,1.66)** ***** | **1.12(1.10,1.13)** ***** |
| **FCRD to Death** | **ref** | **1.11(1.07,1.15)** ***** | **1.30(1.24,1.36)** ***** | **1.06(1.05,1.07)** ***** |
| **CRM to Death** | **ref** | **1.11(1.01,1.22)** ***** | **1.27(1.13,1.41)** ***** | **1.06(1.03,1.08)** ***** |
| **360 days** |  |  |  |  |
| **Health to FCRD** | **ref** | **1.25(1.24,1.27)** ***** | **1.51(1.48,1.53)** ***** | **1.10(1.09,1.10)** ***** |
| **Health to Death** | **ref** | **1.09(1.05,1.13)** ***** | **1.06(1.01,1.12)** ***** | **1.02(1.01,1.03)** ***** |
| **FCRD to CRM** | **ref** | **1.38(1.32,1.45)** ***** | **1.57(1.49,1.66)** ***** | **1.12(1.10,1.13)** ***** |
| **FCRD to Death** | **ref** | **1.11(1.07,1.15)** ***** | **1.29(1.23,1.36)** ***** | **1.06(1.05,1.07)** ***** |
| **CRM to Death** | **ref** | **1.11(1.01,1.22)** ***** | **1.26(1.13,1.41)** ***** | **1.06(1.03,1.08)** ***** |

Note: Models2 were adjusted for age, ethnicity, BMI, education level, TDI, physical activity, dietary patterns, smoking status, alcohol intake status. Models1 were adjusted for age, ethnicity. TDI: Townsend deprivation index, AL: Allostatic load, FCRD: first cardio-renal disease, CRM: cardio-renal multimorbidity, CVD: cardiovascular disease, CKD: chronic kidney disease.

**S10** **Table Association between AL and disease progression after excluding the cardiovascular domain from AL construction.**

|  | **AL** | | | |
| --- | --- | --- | --- | --- |
|  | **Low** | **Mid** | **High** | **Per score** |
| **Health to CVD** | **ref** | **1.16(1.14,1.17)** | **1.28(1.25,1.31)** | **1.06(1.06,1.07)** |
| **Health to CKD** | **ref** | **1.46(1.41,1.51)** | **1.78(1.68,1.89)** | **1.17(1.16,1.18)** |
| **FCRD to Death** | **ref** | **1.10(1.06,1.14)** | **1.17(1.10,1.26)** | **1.05(1.03,1.06)** |
| **CVD to CRM** | **ref** | **1.40(1.33,1.48)** | **1.77(1.61,1.94)** | **1.16(1.14,1.18)** |
| **CKD to CRM** | **ref** | **1.02(0.95,1.10)** | **1.11(0.98,1.25)** | **1.01(0.99,1.04)** |
| **CVD to Death** | **ref** | **1.18(1.13,1.22)** | **1.29(1.20,1.39)** | **1.07(1.05,1.08)** |
| **CKD to Death** | **ref** | **1.07(0.98,1.17)** | **1.32(1.14,1.52)** | **1.06(1.03,1.09)** |
| **CRM to Death** | **ref** | **1.06(0.95,1.18)** | **1.32(1.11,1.56)** | **1.07(1.03,1.10)** |

Note: Models2 were adjusted for age, ethnicity, BMI, education level, TDI, physical activity, dietary patterns, smoking status, alcohol intake status. Models1 were adjusted for age, ethnicity. TDI: Townsend deprivation index, AL: Allostatic load, FCRD: first cardio-renal disease, CRM: cardio-renal multimorbidity, CVD: cardiovascular disease, CKD: chronic kidney disease.

**S11** **Table Alternative clinical thresholds for biomarkers in AL construction.**

| **Domain** | **Variables** | **Age (years)** | **Clinical thresholds** |
| --- | --- | --- | --- |
| Metabolic | Glucose (mmol/L) | – | 7.0 |
|  | Cholesterol (mmol/L) | – | >6.2 |
|  | HDL cholesterol (mmol/L) | – | <1.3 |
|  | Glycated haemoglobin (HbA1c) (mmol/mol) | – | 48 |
|  | IGF-1 (nmol/L) | – |  |
|  |  | <41 | >30.3 |
|  |  | 41-45 | >29.1 |
|  |  | 46-50 | >28.6 |
|  |  | 51-55 | >27.5 |
|  |  | 56-60 | >25.4 |
|  |  | 61-65 | >23.1 |
|  |  | 66-70 | >21.5 |
|  |  | >70 | >21.0 |
|  | Waist circumference/Hip circumference | – | >0.85 |
|  | BMI (kg/m) | – | >30 |
| Inflammatory | C-reactive protein (mg/L) | – | 3 |
| Cardiovascular | Systolic blood pressure (SBP, mmHg) | – | 140 |
|  | Diastolic blood pressure (DBP, mmHg) | – | 90 |

**S12 Table Association between AL and disease progression using alternative clinical thresholds for AL construction.**

|  | **AL** | | | |
| --- | --- | --- | --- | --- |
|  | **Low** | **Mid** | **High** | **Per score** |
| **Health to FCRD** | **ref** | **1.45(1.44,1.47)** | **1.83(1.80,1.86)** | **1.17(1.16,1.17)** |
| **Health to Death** | **ref** | **1.15(1.11,1.19)** | **1.17(1.10,1.23)** | **1.05(1.04,1.07)** |
| **FCRD to CRM** | **ref** | **1.37(1.30,1.44)** | **1.95(1.84,2.06)** | **1.18(1.16,1.20)** |
| **FCRD to Death** | **ref** | **1.15(1.11,1.20)** | **1.39(1.32,1.46)** | **1.09(1.07,1.10)** |
| **CRM to Death** | **ref** | **1.17(1.05,1.30)** | **1.39(1.23,1.56)** | **1.09(1.06,1.12)** |

Note: Models2 were adjusted for age, ethnicity, BMI, education level, TDI, physical activity, dietary patterns, smoking status, alcohol intake status. Models1 were adjusted for age, ethnicity. TDI: Townsend deprivation index, AL: Allostatic load, FCRD: first cardio-renal disease, CRM: cardio-renal multimorbidity, CVD: cardiovascular disease, CKD: chronic kidney disease.
